# Lived experience of pilonidal sinus disease: Systematic review and meta‐ethnography

**DOI:** 10.1111/codi.17295

**Published:** 2025-01-14

**Authors:** Kelsey Aimar, Daniel M. Baker, Elizabeth Li, Matthew J. Lee

**Affiliations:** ^1^ Department of Colorectal Surgery University Hospitals Birmingham NHS Foundation Trust Birmingham UK; ^2^ Leeds Teaching Hospitals NHS Trust Leeds UK; ^3^ University of Birmingham Birmingham UK; ^4^ Institute for Applied Health Research, College of Medical and Dental Sciences University of Birmingham Birmingham UK

**Keywords:** pilonidal disease, proctology, qualitative research, systematic review

## Abstract

**Aim:**

Pilonidal sinus disease (PSD) poses significant treatment challenges due to a lack of consensus on the diverse range of surgical approaches routinely employed, prompting a renewed focus on the patient experience. The aim of this study was to explore the lived experience of patients with PSD to better inform future person‐centred treatment.

**Method:**

A systematic review was performed to identify papers reporting qualitative studies on the lived experience of PSD. The MEDLINE, EMBASE and CINAHL databases were searched, using a predefined search strategy. Studies were dual screened at each stage, with conflicts resolved by a third reviewer. Analytical frameworks were extracted, along with supporting quotes. A meta‐ethnographic approach was used to systemically compare and synthesize frameworks in line with the eMERGe meta‐ethnography protocol. The study was registered on PROSPERO (CRD42024495608).

**Results:**

Four full texts covering three studies were included. Three key themes emerged: (1) disruption to activities of daily living; (2) impact on psychological well‐being; (3) navigating healthcare. Reduction of physical activity was patient‐led, owing to fears of exacerbating symptoms and wound complications. PSD had a complex influence on self‐perception and emotional state, leading to changed relationships with others. This was largely driven by the forced reliance on others for wound care. The final theme highlighted concerns regarding unexpected disease course and outcomes stemming from a lack of patient awareness of PSD.

**Conclusion:**

This study informs a more sophisticated understanding of the experience of individuals living with PSD and has identified recommendations that should guide future clinical practice and research.

**What does this paper add to the literature?**

This paper offers an initial qualitative insight into the nuanced relationship between pilonidal sinus disease and those affected by it. It highlights key concepts to further our understanding of patient‐important outcomes in a research field traditionally focused on surgeon‐driven results and in which the efficacy of interventions appears equivocal.

## INTRODUCTION

Pilonidal sinus disease (PSD) is a condition affecting the midline natal cleft—encompassing a spectrum of symptoms ranging from asymptomatic pits to acutely painful abscesses [1, 2]. Risk factors for PSD include hirsutism, male gender, higher body mass index and habitual prolonged sitting [3]. Caucasian men have been found to have higher incidence, possibly due to differences in hair growth patterns between ethnicities [4, 5, 6]. The condition predominantly affects young adults, typically manifesting in the second decade of life [7, 8]. Men have a higher incidence, being affected two to three times more often than women. In the UK, PSD has been reported to account for over 13 000 hospital admissions annually [9]. The disease produces a large burden on healthcare services, but also to the wider economy due to the typical demographic affected.

PSD poses significant challenges in treatment due to a lack of consensus on the optimal intervention [10]. There are a variety of trialled interventions which range from minimally invasive techniques to natal cleft excision with reconstruction. However, due to heterogeneity within the literature there is a range of reported outcomes with lack of clarity over patient‐reported outcomes [11]. In fact, the current literature is marked by a focus on technical aspects of surgical management and wound care, reflecting a pressing need to also spotlight the pervasive patient experiences associated with the disease. Recognizing this gap, efforts have been directed at synthesizing qualitative evidence on PSD, thereby enabling comparative analysis across multiple studies to derive novel insights into patient experiences.

The aim of this study was to systematically review the lived experiences of patients with PSD. Our objectives were to identify the gaps in knowledge and the unmet needs of clinical treatments alongside the patient experience.

## METHOD

### Overview

This meta‐ethnography of qualitative studies focused on understanding patients' experiences with PSD to better inform the development of patient‐centred intervention and enhance satisfaction with disease management. Meta‐ethnography, a qualitative synthesis methodology developed by Noblit and Hare in 1988, was chosen for its ability to transcend the findings of single studies and develop substantive new interpretations [12]. Unlike traditional literature reviews, meta‐ethnography systematically compares and synthesizes authors' interpretations from primary studies to create new insights and theories that enrich understanding beyond mere aggregation. The method involves comparing key concepts across primary studies and evaluating their relationships, classified into three types: (1) accounts which are directly comparable give rise to ‘reciprocal’ translations; (2) accounts which are in opposition give rise to ‘refutational’ translations; (3) accounts which collectively present a cohesive narrative of the research topic generate a ‘line‐of‐argument’ synthesis [13]. This approach preserves the interpretive properties of the original data, ensuring depth and context while achieving a holistic understanding of the impact of PSD on patients. Our study adhered to a protocol devised originally by Noblit and Hare and reasserted in the eMERGe guidelines supported by the EQUATOR Network for standardized reporting (Supplement S1) [14].

### Search strategy

A systematic search of MEDLINE, EMBASE and CINAHL databases was performed to identify original qualitative papers. A search strategy was developed which included relevant MeSH terms and keywords. Search terms included ‘qualitative’, ‘pilonidal’, ‘pilonidal sinus’, and an example of search strategies is presented in Supplement S2. Searches were conducted with the support of the systematic search service at the Royal College of Surgeons of England. The project is registered on PROSPERO (CRD42024495608) and is reported in line with PRISMA guidance [15].

### Selection process

Search results were loaded into the Covidence systematic review software (Veritas Health Innovation, Australia). Abstracts were dual screened for eligibility (KA/DB), and relevant full texts retrieved. Disagreements were arbitrated by ML. This process was repeated for full texts.

### Risk of bias assessment

The original qualitative studies identified from the study selection process were subjected to critical appraisal using the Joanna Briggs Institute Critical Appraisal Checklist for Qualitative Research (Supplement S3) [16]. This was performed independently by two researchers (KA/DB).

### Data collection

Study characteristics were recorded in an Excel database (Microsoft, CA), including authorship, country of study, publication year, methodological approach, participant demographics and inclusion criteria (Table 1). Each article was subjected to multiple comprehensive readings to extract key themes, second‐order constructs (the original authors' interpretations based on the participants' own words) and participant quotes, all of which were categorized and documented. After identifying common themes across the primary studies, a comparative analysis was performed to highlight similarities and differences among the original findings and define how the studies relate to one another.

**TABLE 1:** Summary of the included studies.

First author	Country	Year	Theoretical approach	Total patients interviewed	Sex (male)	Age range (years)	Inclusion criteria
Bradley	UK	2010	Phenomenological	3	2	18–30	Surgery for PSD and a chronic (>4 months) nonhealing wound
Strong	UK	2021	Framework analysis	20 (baseline), 13 (follow‐up)	14	20–64	Elective surgery for symptomatic PSD
Stewart	Australia	2011, 2012	Interpretive description	11	7	17–39	Postsurgical acute or chronic PSD wound

Abbreviation: PSD, pilonidal sinus disease.

### Synthesis methods

A critical synthesis was conducted to interpretively translate findings into third‐order constructs (the researcher's interpretations of the original authors' interpretations), thereby developing a deeper understanding of PSD from the individual studies. This was a collaborative and reiterative process as interpretations were negotiated by all members of the team with concurrent careful rereadings of the primary research to ensure our constructs remained grounded in the context of the original studies.

## RESULTS

### Study selection

**FIGURE 1:**
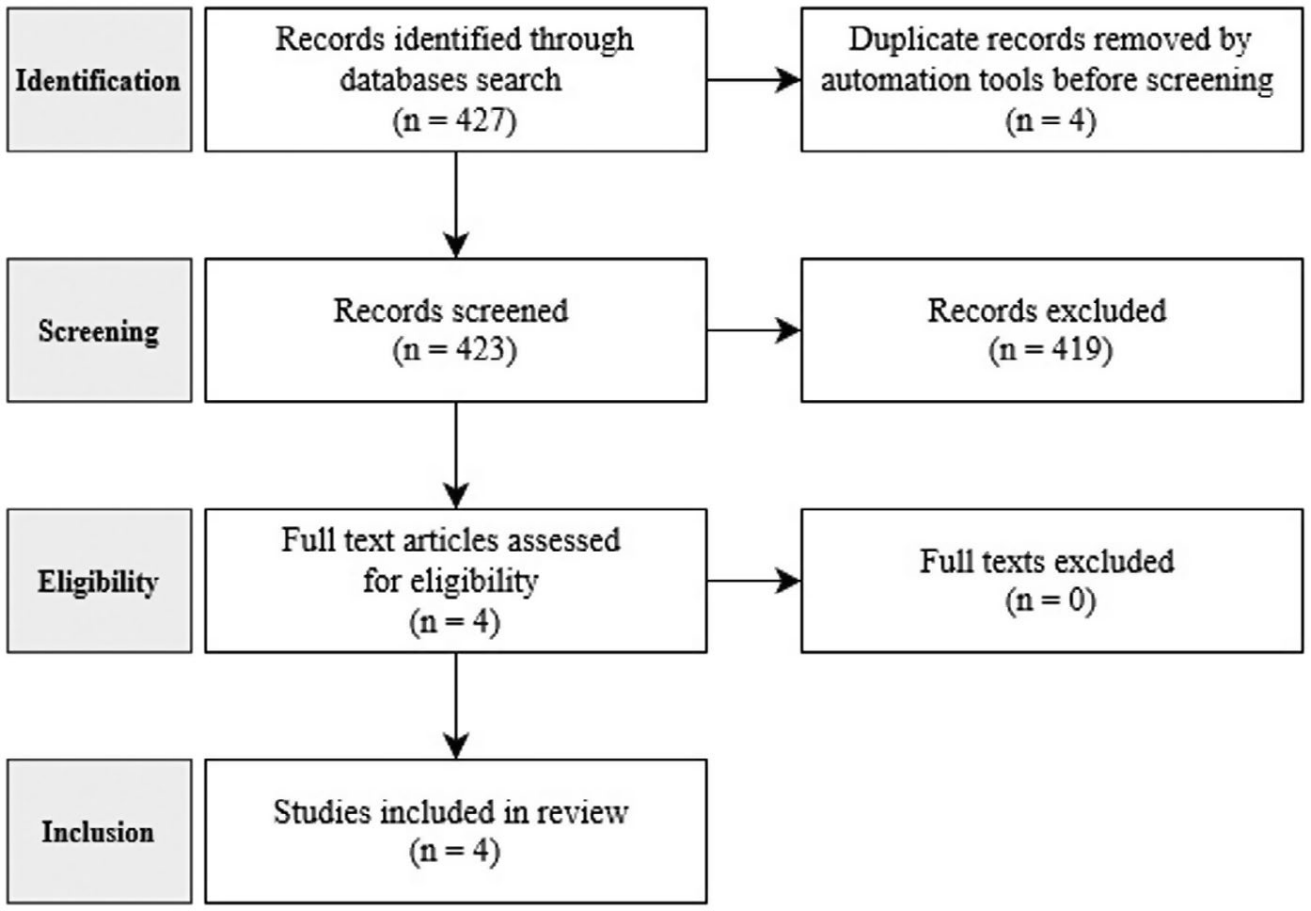
PRISMA flow diagram.

Searches identified 427 papers, of which four were identified and removed as duplicate entries by the Covidence systematic review software (Veritas Health Innovation, Australia). Reports were screened in two stages (Figure 1). In the first stage, 423 abstracts were read and four meeting the inclusion criteria were selected for further eligibility screening. On close reading of the full texts in the second stage of screening all four papers were deemed to have met the inclusion criteria and were synthesized according to the eMERGe meta‐ethnography protocol [14].

### Study characteristics

This meta‐ethnographic synthesis included four articles published between 2010 and 2021 [17, 18, 19, 20]. Notably, two of the articles [19, 20] are reports of the same study; however, they are not duplicates per se—they are published in different journals and use unique participant quotes but express identical themes and second‐order interpretations. We have considered these two papers as one study as they are drawn from the same cohort, reporting different aspects of analysis. Table 1 presents a summary of the three primary qualitative studies that were analysed. Two studies were conducted in the UK and one in Australia. The number of participants interviewed varied between 3 and 20. Male participants comprised the majority, ranging from 64% to 70% of interviewees. The studies each used a different theoretical approach to analysis (phenomenological, framework analysis and interpretive description), though the context was common across all three—patients with PSD who had undergone at least one surgical intervention.

### How the studies are related

Inclusion criteria and participant demographics were similar across the three articles synthesized in this meta‐ethnographic study, thereby facilitating direct comparison of the accounts and giving rise to reciprocal translations. The original studies communicated similar ideas pertaining to the impact of PSD on participants' physical and psychological health, as well as the lack of participant knowledge or education on the disease or its treatments (Table 2). Of note, three articles [17, 19, 20] delved into the lived experience and psychosocial burden of PSD, while another [18] examined patient decision‐making and regret regarding surgical treatment. Rather than presenting opposing views, their findings complemented each other, contributing to a holistic understanding of life with PSD and supporting a coherent synthesis.

**TABLE 2:** Summary of themes reported in the included studies.

Bradley (2010)	Strong (2021)	Stewart (2011, 2012)
Active lifestyle	Health threat	Adaption: Learning to live with the wound Difficulty living with the wound Living life despite the wound Perception: Embarrassment Lack of understanding Changed body image Control: Loss of control Gaining control
Noticing symptoms	Presentation of choice	
Seeking help	Presentation and interpretation of options	
Lifestyle changes	Preference construction	
Demands on time	Decision	
Perceptions of care	Consolidation	
Coping mechanisms	Intervention coherence	
Fluctuating emotions	Opportunity costs	
	Perceived effectiveness	
	Self‐efficacy	

### Critical synthesis

**FIGURE 2:**
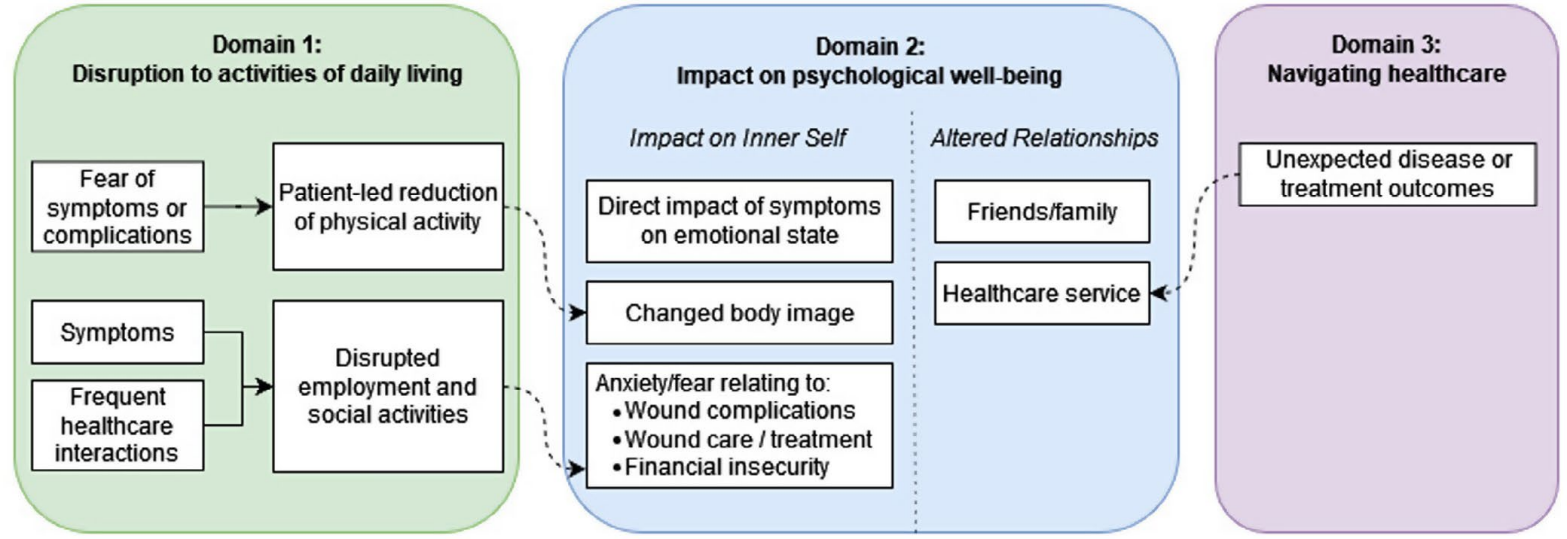
Conceptual framework. Each domain is visually distinguished by a different background colour: green (left) for domain 1 (Disruption to activities of daily living), blue (middle) for domain 2 (Impact on psychological well‐being), purple (right) for domain 3 (Navigating healthcare). Solid lines illustrate direct connections within a domain, while dashed lines link concepts across different domains.

Critical synthesis of the original studies resulted in the development of three key domains (Figure 2) which help to deepen our understanding of participants' lived experiences of PSD: (1) disruption to activities of daily living; (2) impact on psychological well‐being; (3) navigating healthcare. A full collection of participant quotes, organized by domain, is presented in Supplement S4.

#### Domain 1: Disruption to activities of daily living

On surface‐level interrogation of the primary articles, the impact of PSD on participants' activities of daily living was readily apparent. Participant quotes uncovered notable patterns relating to life disruption caused by PSD (Table 3). Reduction of physical activity was not based on clinician advice but was patient‐led, owing to fears of exacerbating wound symptoms such as pain and bleeding or wanting to avoid wound complications such as delayed healing and infection:

I'm scared of lifting things in case I do damage to it. … I find it hard sometimes to bend—you know—to do my shoelaces. I don't want to stretch in case I do anything 'cause it's been that long and I just want it over and done with. … Even when I'm driving, I'm a bit nervous. I don't drive for far distances in case I sit too long. [17]

**TABLE 3:** Emergent themes and subthemes with sample quotes.

Theme	Subtheme	Sample quote
1. Disruption of activities of daily living	Participant‐led reduction of physical activity	It has made me reticent to engage in some activities … exercise and things like that … through the pain and discomfort, and also the chance of sort of popping the cyst … [18]
		The pilonidal sinus has disrupted my life quite a bit, mainly work and activities that I normally enjoy doing. I couldn't go to the gym and couldn't do any exercise and just the sitting was painful. I had continual pain. I used to go to the gym or go for a ride as it helps to reduce my stress. I have been afraid to do weights because I thought that straining my body might reopen the wound. I really haven't done any exercise apart from walking around in my daily routine. [19]
	Disruption to employment and social activities	‘It's a pain in the neck, you know, having it for, like, 9 months I've just had kinda had enough for now and also, with what I do with training weekends, I find sometimes I'm not allowed to attend them in case of infection and what not; plus, you know, keeping on opening up.’ [Researcher]: ‘And how did you feel about that?’ [Participant]: ‘I wasn't happy about it because I wanted to get involved with everything that was going on.’ [17]
		At first I, [the consultant] sort of said, oh you might be back in a … couple of weeks and then when my friend said oh, 12 weeks for this open wound to heal, I thought … I can't take that long off work. I can't afford it. [18]
2. Impact on psychological well‐being	Emotional toll	I was really depressed because I was so young and why did I have to go through all this. I started getting angry and aggressive mainly at myself and it was real hard and there were nights where I would literally cry myself to sleep. I was in such a bad space and I didn't know how to deal with it. I couldn't talk to anyone because I felt embarrassed and it made me feel soft [weak]. [19]
		I don't have my typical release, which is going for a run, or going down to the golf course. If you don't have that, you don't have an activity that enables you to just close off. Some people read, some people listen to music; I need to do something physical. I don't have that outlet and it just builds up. I've been on edge quite a bit and blow up because I can't get out and do things and get rid of that build up. [20]
	Altered relationships with others	… my friend had to sort of dress it for me … I was even more embarrassed. [17]
		It affected my partner and our relationship. He was frightened he would hurt me and it was the last thing on my mind because the wound was so sore and when it became OK and wasn't sore anymore it was the fact it was there and the dressing was there. I felt ugly and unattractive. [19]
3. Navigating healthcare	Unexpected disease or treatment outcomes	I was told it was going to get better in 2 weeks, but I never heard as much nonsense. [17]
		I expected to be in [hospital] two or three days, come out and it would be healed within two or three months, happy days, life goes on. In hindsight, if I had of thought that it was going to take as long as what it has done I probably would have put up with the little cyst of the pilonidal sinus. It has been a nightmare. [20]

PSD disrupted employment, social and physical activities with significant secondary effects on participants' well‐being. Reduction of exercise leading to weight gain, or in some cases loss of muscle mass, and was closely related to feelings of depression and pent‐up stress. The idea of taking time off work was related to anxiety of financial insecurity, with one participant stating:

At first I, [the consultant] sort of said, oh you might be back in a … couple of weeks and then when my friend said oh, 12 weeks for this open wound to heal, I thought … I can't take that long off work. I can't afford it. [18]

Notably, disruption to life activities was not solely attributable to PSD symptoms but also occurred because of frequent interactions with health services over prolonged periods of time. This was primarily related to routine wound management in the postoperative setting but was also linked with recurrence of disease which necessitated repeated specialist assessment and treatment.

#### Domain 2: Impact on psychological well‐being

A second key theme was the impact of PSD on individuals' psychological well‐being. This was characterized by complexities relating to changing perceptions of the self and of one's relationships with others. Participants commonly reported personal struggles with depression, shame, embarrassment, anxiety and fear. Although some of this can be accounted for by changes in body habitus leading to altered body image, the symptoms of PSD and even the diagnosis itself contributed to pervasive emotional turmoil. Potentially noticeable symptoms such as bleeding or malodorous discharge were particularly impactful and predominantly gave rise to feelings of embarrassment. Our interpretations of participant quotes across the original studies also revealed a high degree of anxiety around potential complications and fear of treatment and wound care practices such as packing.

PSD had a notable impact on participants' relationships with their friends, family members and healthcare services. The primary factor driving change in interpersonal relationships was the forced reliance on others for ongoing wound care due to the body site affected by the disease:

I think the worst part of it is that you always have to rely on someone else to do, like, a dressing for you … you can't drive cos you can't sit down … you basically you can't do anything. [18]

This fostered feelings of frustration relating to loss of independence and was also strongly linked to embarrassment.

#### Domain 3: Navigating healthcare

Navigating the healthcare system and managing PSD present numerous challenges for patients, as evidenced in the three papers. Participants with acute presentations requiring emergency treatment looked upon their healthcare interaction more favourably than those on waiting lists when referred from primary care. Following surgery for PSD, participants' perceptions of their treatment outcomes were diverse. Some remarked on a smooth process, making such statements as:

It all, all went well. You know there's, there's no reason for me to want to do anything differently. [18]

However, others expressed surprise and anger over unexpected disease or treatment results:

We thought the wound was only going to be a small one, a couple of centimetres … it wasn't, it was 12 cm, and we thought I'd be in and out and healed in a couple of weeks but I wasn't. [19]

Major concerns were noted about participants' lack of awareness of the wound size and healing time required. Participants' poor understanding of PSD and its treatment was most explicitly explored in the article by Strong et al. [18], though it became apparent that this element was consistent across all three studies. Such gaps in knowledge led to increased psychological burden and discontent with the health service. The lack of clear information, alongside the unpredictability of the disease, intensified feelings of uncertainty and anxiety. Across all these original studies there was a call for greater patient support and accurate information‐giving on PSD.

The three domains demonstrated a degree of interconnectedness. Disruption to activities of daily living (domain one) impacted on participants' psychological well‐being (domain two). This interplay of themes specifically pertained to the ‘inner self’ component of domain two, with body image concerns tied to reduced physical activity and fears of financial insecurity linked to work limitations imposed by the disease (Figure 2). Domain three intersected with the ‘altered relationships’ component of domain two, as unexpected disease or treatment outcomes were connected to participants' views on the health service.

### Risk of bias assessment

The original works included in this study exhibited congruity between their stated philosophical perspectives, research methodologies and data analysis. Studies uniformly performed well but failed to report the influence researchers' personal views may have had on the results.

## DISCUSSION AND CONCLUSION

This study identified three key themes that inform a more sophisticated understanding of the experience of individuals living with PSD. Themes one and two explored the impact of the disease on activities of daily living and psychological well‐being, respectively, whereas theme three highlighted inadequacies in patient education on PSD. Research into the physical and mental consequences of chronic wounds in general has emerged in recent years [21, 22, 23], but few studies beyond the three included here have reported on PSD specifically [24, 25]. This study therefore presents an initial qualitative insight into the complex relationship between PSD and the patient population.

PSD imparts a high degree of disruption with sweeping effects on all aspects of daily life including sleep, exercise, employment and social interactions. In this study, reduced engagement in daily activities and exercise was driven by patients themselves, guided by their symptoms and influenced by fears of causing complications. There are no professional guidelines that advise against exercise for those with pilonidal wounds or those recovering from surgery to treat PSD [26]. In fact, emerging research suggests that physical activity may aid wound healing [27, 28]. Maintaining activity could mitigate mental health issues stemming from the prolonged idleness reported in this study [29]. However, reassurance alone is insufficient: patients require clear guidance on when and how to modify activities. Moreover, effective symptom management, especially for severe pain, is vital to enable patients to resume routines [10]. Alongside enhancing analgesic protocols, it is pertinent to reassess the evidence base behind routine wound packing in managing this condition. Current studies advocate for the omission of traditional internal dressings to reduce postoperative pain and enhance patient satisfaction without compromising morbidity or recurrence outcomes [30, 31, 32, 33]. Evidence supports a shared‐care approach to wound management, which allows patients greater control over their treatment, with benefits for both patients and overburdened health services [34].

A critical finding of this study was that participants frequently had poor understanding of PSD and expectations surrounding treatment outcomes. While acknowledging that explaining PSD can be challenging, due to its variable clinical presentation and management, this does not account for participants' gaps in knowledge. Some individuals were unaware of how common the disease is or that it can affect both women and men. Psychological distress mainly arose from lack of understanding regarding healing rates and recurrence. Among a subset of participants, the persistence or recurrence of the disease over months or years elicited surprise, frustration and dissatisfaction with healthcare services. Possible factors contributing to poor understanding include inadequate information provided, difficulties in comprehension and difficulties in remembering what was discussed. Recognized as a critical area of importance, there is an abundance of literature including professional guidelines dedicated to improving communication between healthcare providers and patients [35]. Conveying clear and accurate information is crucial for effective, person‐centred care, as patients' understanding of their illness can substantially influence their recovery and the lived experience within which that takes place.

One strength of this study was that few publications have explored patients' subjective experiences of treatment for PSD, allowing our synthesis to remain firmly grounded in the content of the original texts. Such preservation of familiarity with the primary works, even after tertiary interpretation, is a central tenet of meta‐ethnography: the seminal description of the meta‐ethnographic method by Noblit and Hare was based on just two to six studies [12]. More recent literature has demonstrated the feasibility of synthesizing large numbers of studies; however, this risks losing meaning and accuracy in the iterative process [36, 37, 38]. While some have suggested that the inclusion of approximately 40 articles would achieve a balance between the breadth and depth of data analysed, this remains untested [39]. We must acknowledge such a trade‐off in the context of our study. Constrained by the paucity of research in this area, we consider that the incorporation of only three studies may have left some aspects of the lived experience of PSD unexplored. Specifically, the impact of cultural factors on individuals with PSD was not addressed. All of the included studies were conducted in western cultures and, with the exception of one article, the ethnicities of participants were not reported. Moreover, non‐English publications were excluded during screening due to funding constraints. This limitation could affect the generalizability of our findings and may overlook culturally relevant elements of living with PSD. In light of the scarcity of qualitative research in this area, a rigorous methodology was applied to the existing data to secure reliable results. A key feature of this approach was collaboration of multiple researchers at the critical synthesis phase. Differing levels of experience of qualitative research and of PSD lent diverse perspectives on the content of the primary texts, thereby avoiding one‐sided interpretation.

The findings of this study hold meaningful implications for policy, clinical practice and future research. PSD primarily affects people of working age and is highly disruptive to employment activities [10]. Recommendations to minimize the socioeconomic burden of PSD include providing clearer guidance on activity postsurgery, improving pain management and using minimally invasive approaches with less morbidity and a shorter recovery time than major skin excisional techniques [40]. Navigating the healthcare system was a challenge for patients, especially if actual treatment outcomes did not align with what was expected. Therefore, it is imperative that patient education in clinical practice is improved. Future research priorities in PSD are recommended in a consensus statement informed by the PITSTOP (PIlonidal sinus Treatment: STudying the OPtions) research [40]. Among the priorities is the need for a patient‐reported outcome measure (PROM) that would provide insight into the quality of interventions from patients' perspectives. This study may inform a PSD‐specific PROM by highlighting key concepts and domains for exploration.

## AUTHOR CONTRIBUTIONS

**Kelsey Aimar:** Conceptualization; writing – original draft; formal analysis; data curation; writing – review and editing; investigation; project administration. **Daniel M. Baker:** Conceptualization; investigation; writing – review and editing; formal analysis; writing – original draft. **Elizabeth Li:** Investigation; methodology; writing – review and editing; writing – original draft; supervision. **Matthew J. Lee:** Conceptualization; investigation; writing – original draft; writing – review and editing; methodology; software; project administration; supervision; resources.

## FUNDING INFORMATION

No funding received.

## CONFLICT OF INTEREST STATEMENT

The authors declare no conflicts of interest.

## ETHICS STATEMENT

No ethical approval was required for this meta‐ethnographic study of previously published qualitative research.

## Supporting information


Data S1.

## Data Availability

Data sharing is not applicable to this article as no new data were created or analyzed in this study.
