# Supplementary material for: Lived experience of pilonidal sinus disease: Systematic review and meta‐ethnography
Source: Colorectal Dis. 2025 Jan 14;27(1):e17295. doi: 10.1111/codi.17295 (PMC11732642; doi:10.1111/codi.17295)
Supplement: Supplementary file 1 — Data S1. [file CODI-27-0-s001.docx]

**Supplement 1:** eMERGe meta-ethnography reporting criteria with page numbers of corresponding explanatory notes in the primary article

| Meta-ethnography Phase | eMERGe Reporting Criteria | Page number* |
| --- | --- | --- |
|  |  |  |
| *Phase 1 -* Selecting meta-ethnography and getting started | 1. Rationale and context for the meta-ethnography | 5 |
|  | 2. Aim(s) of the meta-ethnography | 5 |
|  | 3. Focus of the meta-ethnography | 5 |
|  | 4. Rationale for using meta-ethnography | 6 |
| *Phase 2 -* Deciding what is relevant | 5. Search strategy | 7 |
|  | 6. Search processes | 7 |
|  | 7. Selective primary studies | 7 |
|  | 8. Outcome of study selection | 7 |
| *Phase 3 -* Reading included studies | 9. Reading and data extraction approach | 7 |
|  | 10. Presenting characteristics of included studies | 7 |
| *Phase 4 -* Determining how studies are related | 11. Process for determining how studies are related | 8 |
|  | 12. Outcome of relating studies | 8 |
| *Phase 5 -* Translating studies into one another | 13. Process of translating studies | 8 |
|  | 14. Outcome of translation | 9 |
| *Phase 6 -* Synthesizing translations | 15. Synthesis process | 9 |
|  | 16. Outcome of synthesis process | 9 |
| *Phase 7 -* Expressing the synthesis | 17. Summary of findings | 9 |
|  | 18. Strengths, limitations, and reflexivity | 10 |
|  | 19. Recommendations and conclusions | 10 |
| *France EF, Cunningham M, Ring N, Uny I, Duncan EAS, Jepson RG, et al. Improving reporting of meta-ethnography: the eMERGe reporting guidance. BMC Med Res Methodol. 2019;19(1):25. | | |

**Supplement 2:** Search strategy

| **Database:** | **Ovid MEDLINE(R) ALL <1946 to December 11, 2023>** |
| --- | --- |
| **Date:** | **12/12/2023** |
| 1 | Pilonidal Sinus/ |
| 2 | ((pilonid* or sacrococcygeal) adj3 (cyst* or sinus* or abscess* or disease or fistula*)).ti,kw,kf. |
| 3 | ((pilonid* or sacrococcygeal) adj3 (cyst* or sinus* or abscess* or disease or fistula*)).ab. /freq=2 |
| 4 | intergluteal pilonidal.ti,ab,kw,kf. |
| 5 | pilonidal.ti. |
| 6 | (EPSIT or PEPSIT or endoscopic pilonidal sinus treatment).ti,kw,kf. |
| 7 | (VAAPS or video-assisted ablation of pilonidal sinus or (Endoscopic-assisted Pilonidal Irrigation and Cleaning)).ti,kw,kf. |
| 8 | 1 or 2 or 3 or 4 or 5 or 6 or 7 |
| 9 | exp qualitative research/ |
| 10 | exp "Quality of Life"/ |
| 11 | exp Patient Satisfaction/ |
| 12 | (patient* adj3 (experience or experiences)).ti,kw,kf. |
| 13 | (patient* adj3 (experience or experiences)).ab. /freq=2 |
| 14 | qualitative*.ti,kw,kf. |
| 15 | (satisf* or preference* or QoL or "quality of life").ti,kw,kf. |
| 16 | exp "Surveys and Questionnaires"/ |
| 17 | (survey* or questionnaire*).ti,kw,kf. |
| 18 | 9 or 10 or 11 or 12 or 13 or 14 or 15 or 16 or 17 |
| 19 | 8 and 18 |
| 20 | limit 19 to english language |

| **Database:** | **Embase <1974 to 2023 Week 49>** |
| --- | --- |
| **Date:** | **12/12/2023** |
| 1 | Pilonidal Sinus/ |
| 2 | ((pilonid* or sacrococcygeal) adj3 (cyst* or sinus* or abscess* or disease or fistula*)).ti,kw,kf. |
| 3 | ((pilonid* or sacrococcygeal) adj3 (cyst* or sinus* or abscess* or disease or fistula*)).ab. /freq=2 |
| 4 | intergluteal pilonidal.ti,ab,kw,kf. |
| 5 | pilonidal.ti. |
| 6 | (EPSIT or PEPSIT or endoscopic pilonidal sinus treatment).ti,kw,kf. |
| 7 | (VAAPS or video-assisted ablation of pilonidal sinus or (Endoscopic-assisted Pilonidal Irrigation and Cleaning)).ti,kw,kf. |
| 8 | 1 or 2 or 3 or 4 or 5 or 6 or 7 |
| 9 | exp qualitative research/ |
| 10 | exp "quality of life"/ |
| 11 | patient satisfaction/ |
| 12 | (patient* adj3 (experience or experiences)).ti,kw,kf. |
| 13 | (patient* adj3 (experience or experiences)).ab. /freq=2 |
| 14 | qualitative*.ti,kw,kf. |
| 15 | (satisf* or preference* or QoL or "quality of life").ti,kw,kf. |
| 16 | exp questionnaire/ |
| 17 | (survey* or questionnaire*).ti,kw,kf. |
| 18 | 9 or 10 or 11 or 12 or 13 or 14 or 15 or 16 or 17 |
| 19 | 8 and 18 |
| 20 | limit 19 to english language |

**Supplement 3:** Joanna Briggs Institute (JBI) critical appraisal checklist for qualitative research

| Checklist Questions | Yes | No | Unclear | N/A |
| --- | --- | --- | --- | --- |
|  |  |  |  |  |
| 1. Is there congruity between the stated philosophical perspective and the research methodology? | ▢ | ▢ | ▢ | ▢ |
| 2. Is there congruity between the research methodology and the research question or objectives? | ▢ | ▢ | ▢ | ▢ |
| 3. Is there congruity between the research methodology and the methods used to collect data? | ▢ | ▢ | ▢ | ▢ |
| 4. Is there congruity between the research methodology and the representation and analysis of data? | ▢ | ▢ | ▢ | ▢ |
| 5. Is there congruity between the research methodology and the interpretation of results? | ▢ | ▢ | ▢ | ▢ |
| 6. Is there a statement locating the researcher culturally or theoretically? | ▢ | ▢ | ▢ | ▢ |
| 7. Is the influence of the researcher on the research, and vice-versa, addressed? | ▢ | ▢ | ▢ | ▢ |
| 8. Are participants, and their voices, adequately represented? | ▢ | ▢ | ▢ | ▢ |
| 9. Is the research ethical according to current criteria or, for recent studies, and is there evidence of ethical approval by an appropriate body? | ▢ | ▢ | ▢ | ▢ |
| 10. Do the conclusions drawn in the research report flow from the analysis, or interpretation, of the data? | ▢ | ▢ | ▢ | ▢ |

**Supplement 4:** Table 3 with full range of sample quotes

| **TABLE 3. Emergent Themes and Subthemes with Sample Quotes** | | |
| --- | --- | --- |
| Theme | Subtheme | Sample quote |
|  |  |  |
| 1. Disruption of activities of daily living | Participant-led reduction of physical activity | I'm scared of lifting things in case I do damage to it. …I find it hard sometimes to bend - you know - to do my shoelaces. I don't want to stretch in case I do anything 'cause it's been that long and I just want it over and done with. …Even when I'm driving I'm a bit nervous. I don't drive for far distances in case I sit too long. [17]  It has made me reticent to engage in some activities… exercise and things like that… through the pain and discomfort, and also the chance of sort of popping the cyst… [18]  The pilonidal sinus has disrupted my life quite a bit, mainly work and activities that I normally enjoy doing. I couldn't go to the gym and couldn't do any exercise and just the sitting was painful. I had continual pain. I used to go to the gym or go for a ride as it helps to reduce my stress. I have been afraid to do weights because I thought that straining my body might reopen the wound. I really haven't done any exercise apart from walking around in my daily routine. [19] |
|  | Disruption to employment and social activities | Well, I'm not able …I like playing sport, maybe golf and I'm not able to do that there. I'm going on holiday. I'll not be able to enjoy …I won't be able to participate going to the pool, playing any sport on the beach. …It gets you down that way, and since I've had it I've put on weight 'cause of it. I never do no exercise whatsoever; it gets me down and it just is a nightmare. ...It is like ruining many things. [17]  “It's a pain in the neck, you know, having it for, like, 9 months I've just had kinda had enough for now and also, with what I do with training weekends, I find sometimes I'm not allowed to attend them in case of infection and what not; plus, you know, keeping on opening up." Researcher: "And how did you feel about that?" Participant: "I wasn't happy about it because I wanted to get involved with everything that was going on.” [17]  It's frustrating having to go to the hospital. If I go out on a Saturday night it means I know I have to get up on a Sunday morning to go to the hospital. It's a bit of a nuisance, so it is. But I don't mind as long as it's going to do the job; but it's upsetting me. [17]  My life in that time has been very different to what it was before. …I've basically been at the health centre at least once a week, some weeks every day and having to leave work and what not and rush there and back, and not having been able to settle in your lunch hour ... I feel I have been on the go for a few years not having time. [17]  At first I, [the consultant] sort of said, oh you might be back in a… couple of weeks and then when my friend said oh, 12 weeks for this open wound to heal, I thought… I can't take that long off work. I can't afford it [18]  One day when the nurses were running late and I couldn't wait any longer, I had to dress the wound myself and went off to work and it was fine. I'm working in a toy shop and lifting heavy boxes and again the weight of the boxes didn't really affect it either. [19] |
| 2. Impact on psychological well-being | Emotional toll | I was really depressed because I was so young and why did I have to go through all this. I started getting angry and aggressive mainly at myself and it was real hard and there were nights where I would literally cry myself to sleep. I was in such a bad space and I didn't know how to deal with it. I couldn't talk to anyone because I felt embarrassed and it made me feel soft (weak). [19]  I was very emotional. I think it had a lot to do with the pain killers I was taking. I was very depressed yeah very … Probably only for the first two to three weeks after getting out of the hospital. [20]  It took a fair few years to have it checked out. I was a bit embarrassed. I didn’t want anyone to look at it until it actually started discharging. No one at work had ever heard about it. I described it as being on my lower back like a cyst or a growth... I'm still worried that when I go back to work something embarrassing might happen and it's going to start bleeding or something. [19]  I never actually made it a big deal because it never occurred to me how serious the situation is until recently after having so many surgeries. It really hit me before I went into the theatre for the operation that this is the fifth time that I've had it and I think that there's a problem and I just couldn't take it. I felt really scared because I've been through it so many times. It's been with me for nearly four years now and it's not going away so it just gets to you. In the beginning I was younger and didn't really care. Now it just gets scary, but I've come to live with it. It's just part of my life. [19]  I don't have my typical release, which is going for a run, or going down to the golf course. If you don't have that, you don't have an activity that enables you to just close off. Some people read, some people listen to music; I need to do something physical. I don't have that outlet and it just builds up. I've been on edge quite a bit and blow up because I can't get out and do things and get rid of that build up. [20]  I was sleeping a lot. I was feeling depressed. I actually lost eight kilos in a period of three weeks and so just that as well made me feel depressed. [20]  It really got depressing last year because I put on a lot of weight and clothes that I'd want to wear I couldn't fit into. It's not that I'm overeating, but it's because I can't get out and do any exercises. [20]  I get quite depressed. It's embarrassing. I wake up and go I don't think I can do this anymore emotionally. [20]  It has not just affected me physically, it has affected me mentally. The pain and seepage have been ridiculous from the word dot [the beginning]. It's just a nasty, nasty thing to have and I wouldn't wish it on my worst enemy. [20] |
|  | Altered relationships with others | …Basically I was scared stiff of having, it wasn't the operation … it was just the packing. I was frightened. I didn't want to go through all that pain again and I was trying to tell a friend about it and what not, 'cause I was really up to high doh you know. She said oh you'll be all right and what not, sure I'll come up and visit you and I thought nah, there's not much point talking to her she just doesn't understand. So right now it's just the fear of having to go back into hospital again and then packing it instead of stitching it. That's my biggest fear. [17]  There's him again to get it done again … I felt as if I was annoying everybody, like, because I had to go up every day. [17]  My friend had to sort of dress it for me… I was even more embarrassed. [17]  I think the worst part of it is that you always have to rely on someone else to do, like, a dressing for you… you can't drive cos you can't sit down… you basically you can't do anything. [18]  It affected my partner and our relationship. He was frightened he would hurt me and it was the last thing on my mind because the wound was so sore and when it became ok and wasn't sore anymore it was the fact it was there and the dressing was there. I felt ugly and unattractive. [19]  It absolutely affects your independence because you need to sit around in the morning and wait for them to come. You can't organise anything and due to the nature of the nurses they can't tell you whether they will be here at a specific time. [19]  My mum had to overcome a few things to help me with the dressings. She's got a very weak stomach, so to have to pack the wound was quite an ordeal for her but she came through and I'm quite proud of her. [19] |
| 3. Navigating Healthcare | Unexpected disease or treatment outcomes | I was told it was going to get better in 2 weeks, but I never heard as much nonsense. [17]  It's just every month goes by and you give your payment and you think next month it's going to be healed, …but one month after the other it just goes along. [17]  I was told initially, 'Oh that could be it, and then it might go away'… but once you get it once, that's it: it's coming back… If I was a bit more aware of that I would have probably started to look into the surgeries quicker. [18]  We thought the wound was only going to be a small one, a couple of centimetres… it wasn't, it was 12 cm and we thought I'd be in and out and healed in a couple of weeks but I wasn't. [19]  I expected to be in [hospital] two or three days, come out and it would be healed within two or three months, happy days, life goes on. In hindsight, if I had of thought that it was going to take as long as what it has done I probably would have put up with the little cyst of the pilonidal sinus. It has been a nightmare. [20] |
